# The First Human Vulvar Intraepithelial Neoplasia Cell Line with Naturally Infected Episomal HPV18 Genome

**DOI:** 10.3390/v14092054

**Published:** 2022-09-16

**Authors:** Ming Wu, Xiu Zhang, Yiyi Kang, Yaqi Zhu, Zhaoyu Su, Jun Liu, Wei Zhang, Hong Chen, Hui Li

**Affiliations:** 1 State Key Laboratory of Virology, Institute of Medical Virology, School of Basic Medical Sciences, Wuhan University Taikang Medical School, Wuhan 430071, China; 2 Clinical Laboratory, Hubei Maternal and Child Health Hospital, Wuhan 430070, China; 3 Department of Obstetrics and Gynecology, Zhongnan Hospital of Wuhan University, Wuhan 430071, China

**Keywords:** conditional reprogramming (CR), vulvar intraepithelial neoplasia (VIN) cells, episomal HPV18, air-liquid interface (ALI) culture, life cycle

## Abstract

Persistent infection with high-risk HPV leads to cervical cancers and other anogenital cancers and head and neck carcinomas in both men and women. There is no effective drug fortreating HPV infection and HPV-associated carcinomas, largely due to a lack of models of natural HPV infection and the complexity of the HPV life cycle. There are no available cell lines from vulvar, anal, or penile lesions and cancers in the field. In this study, we established the first human cell line from vulvar intraepithelial neoplasia (VIN) with naturally infected HPV18 by conditional reprogramming (CR) method. Our data demonstrated that VIN cells possessed different biological characteristics and diploid karyotypes from HPV18-positive cancer cells (HeLa). Then, we determined that VIN cells contained episomal HPV18 using approaches including the ratio of HPV E2_copy_/E7_copy_, rolling cycle amplification, and sequencing. The VIN cells expressed squamous epithelium-specific markers that are different from HeLa cells, a cervical adenocarcinoma cell line. When cultured under 3D air–liquid interface (ALI) system, we observed the expression of both early and late differentiation markers involucrin and filaggrin. Most importantly, we were able to detect the expression of viral late gene L1 in the cornified layer of ALI 3D culture derived from VIN cells, suggesting quite different HPV genomic status from cancer cells. We also observed progeny viral particles under transmission electron microscopy (TEM) in ALI 3D cultures, confirming the episomal HPV18 genome and active viral life cycle in the new cell line. To our knowledge, this is the first human VIN cell line with naturally infected HPV18 genome and provides a valuable model for HPV biology studies, HPV-associated cancer initiation and progression, and drug-screening platforms.

## 1. Introduction

There are more than 200 subtypes of human papillomaviruses (HPVs) identified within the human population [1]. However, infection with different HPV types results in varyingsubclinical and clinical manifestations [2]. Persistent infection with high-risk (HR) HPVs leads not only to cervical cancers in women but also to anogenital cancers and head and neck carcinomas in both men and women [3,4]. Although a prophylactic HPV vaccine was introduced in 2006, the availability and hesitancy of vaccination have impeded its protective coverage of the population [5]. To date, there is no effective drug for treating HPV infection and HPV-associated carcinoma due to the complexity of the HPV life cycle and the lack of appropriate naturally HPV-infected in vitro models. HPV infection-associated neoplasias and cancers in cervix, anus, penis, and vulva will remain a significant health burden in the upcoming 10–30 years. 

Cervical cancer studies have relied on a limited number of cancer cell lines, e.g., HeLa (integrated HPV18), SiHa (integrated HPV16), CaSki (integrated HPV16), and C33A (mutant p53 and HPV negative). After having been heavily and widely used and cultured in vitro for decades, these cell lines can hardly represent patient-derived cancer biology. In the natural course of HPV infection at the cervix, viruses can be cleared by the immune system at early stages, and neoplasia can regress before progression to invasive cancer [2]. In comparison with the stage of cancer, precancerous lesions should be critical for more comprehensive studies since HPVs with natural life cycles interact with host cells and eventually transform the host cells for cancer initiation and progression. Cervical precancerous lesions are classified into cervical intraepithelial neoplasia (CIN) I-III according to different histology stages. HR-HPVs, most often HPV16, are associated with lesions or cancers of the vulva, anus, and penis that consist of stratified squamous epithelium [6,7]. The majority of vulvar and vaginal cancers (>75%) and high-grade vulvar intraepithelial neoplasias (VIN) II/III are HPV16 positive, but a range of LR-HPVs aredetected in low-grade vulvar lesions (VIN I) [8]. Vulvar and anal high-grade dysplasia and cancers often occur in women with a previous history of HPV-related cervical lesions or cancers [9], suggesting that dysplastic cells may self-transplant from the cervix to other genital areas [10]. Unfortunately, only two cell lines from CIN lesions, W12 with HPV16 and CIN 612 with HPV31, have been established in the past 30 years [11,12]. So far, there is no cell line from human lesions and cancers in anus, penis, or vulva available for studies.

Due to the availability of specimens and cells, human foreskin keratinocytes (HFKs) have been used for HPV biology and the development of cervical cancer [3,13]. However, HPV genotypes are geographically different and also vary in HPV-associated precancer lesions and cancers [2]. The outcome of HPV infection most likely depends on the inherent properties of its natural host cells in which HPV can amplify and go through its life cycle [2]. Therefore, it is of great importance to establish cell lines from anatomically distinct lesions and cancers for HPV biology, cancer initiation and progression, and personalized treatment.

In this study, we established the first human cell line from the vulvar intraepithelial neoplasia (VIN) by conditional reprogramming (CR) [14,15]. We demonstrated that VIN cells proliferated indefinitely under CR condition and that these cells maintained the different biological characteristics (differentiation and response to DNA damage) and did not have transforming property in vitro, in contrast with HeLa cells, an HPV18-positive cervical cancer cell line. Rolling circle amplification (RCA) and sequencing validated that VIN cells contained an episomal HPV18 genome. Previous studies suggested that CR cells possess the lineage differentiation potential [14,16,17], and our data also indicated that CR-VIN cells formed a differentiated stratified epithelium under air–liquid (ALI) 3D culture, which supports the natural HPV life cycle. Most importantly, we found that HPV18 was amplified, that L1 capsid protein was expressed in cornified layers, and that viral particles were assembled during the differentiation of VIN cells in ALI 3D cultures. These results suggested that these naturally infected HPV18 cells maintained the episomal HPV genome and allowed HPV18 replication under ALI 3D culture. VIN cell-derived ALI 3D culture provides a valuable model for studying HPV biology and HPV-associated cancer.

## 2. Materials and Methods

### 2.1. Cell Isolation and Cell Culture

This study was carried out according to the Declaration of Helsinki and approved by the Institutional Review Board of Zhongnan Hospital of Wuhan University. Written informed consent was obtained from the patients. Colposcopy was performed, and small biopsies were collected from the cervix and vulva. The vulvar biopsies showed vulvar intraepitheilial neoplasia (VIN) III on histological examination, and one portion of the same biopsy was sent for primary cell culture as previously described [14,15,18]. In brief, sample was minced and digested with collagenase (StemCell, Canada) and trypsin. Dispersed single cells were co-cultured with feeder cells (irradiated mouse fibroblast 3T3 cells) in medium supplemented with ROCK inhibitor as described previously [15,18]. Human normal vulvar epithelial cells (NVECs) were established and cultured in a previous study [18]. HeLa cells were maintained in DMEM supplemented with 10% FBS. Cells were cultured under the condition of 5% CO_2_ at 37 °C. The cell growth curve was plotted as accumulated population doublings (PDs) versus time (days) [18].

### 2.2. Short Tandem Repeat (STR) Analysis

DNA was extracted from VIN cells with TIANamp Genomic DNA Kit (Tiangen Biotech, Beijing, China) and sent to Shanghai Jiyi Biotechnology Co., Ltd. (Shanghai, China) for STR analysis. Twenty-two highly polymorphic loci of genes (including X chromosome) were analyzed and listed.

### 2.3. Soft Agar Assay

Soft agar assay was performed as described previously [18].

### 2.4. Matrigel Three-Dimensional (3D) Culture

Single-cell suspensions of VIN cells were seeded in keratinocyte growth medium (ThermoFisher Scientific, Waltham, MA, USA) containing 5% precooling matrigel (BD Biosciences, Waltham, MA, USA). The matrigel cultures were cultured in a humidified incubator with 5% CO_2_ at 37 °C and harvested after 7 days for DAPI staining or DAB staining as previously described [17,19].

### 2.5. Karyotype Analysis

After the removal of feed cells with EDTA and PBS, VIN cells were sent to Hangzhou Kautaispectrum Biotechnology Co., LTD, (Hangzhou, China) for karyotype analysis. At least twenty metaphase spreads of VIN cells were counted and photographed under the microscope.

### 2.6. Rolling Circle Amplification (RCA) and Enzyme Digestion Reaction

DNA was extracted from VIN cells and used for rolling circle amplification (RCA) (GE Healthcare Life Science, Pittsburgh, PA, USA) according to the manufacturer’s instructions. RCA is an isothermal amplification in which a circular DNA is set as the template and a short DNA primer is amplified to form a long single-stranded DNA. After amplification, RCA products were digested by restriction enzymes (New England Biolabs, Ipswich, MA, USA). The digested fragments were sent to Sangon Biotech (Shanghai, China) for sequencing.

### 2.7. Construction of Plasmid 

PUC19 vector (Sangon Biotech, Shanghai, China) was linearized by *Eco*RI (New England Biolabs). HPV18 E7 and E2 were amplified by PCR (primer sets listed in Table 1: HPV18 E2 (fl)-F/HPV18 E2 (fl)-R and HPV18 E7 (fl)-F/HPV18 E7 (fl)-R and constructed into linearized pUC19 by 2 × MultiF Seamless Assembly Mix (RK21020, ABclonal Technology, Wuhan, China) [20]. The recombinant plasmid pUC19-HPV18 E2/E7 was verified by sequencing.

### 2.8. PCR and Quantitative RT-PCR

DNA was isolated from cells, and PCR amplification was carried out with specificprimers (Table 1). RNA was extracted by TRIZOL (ThermoFisher Scientific, Waltham, MA, USA), and cDNA was achieved using PrimeScript™ RT Master Mix (Takara Bio Inc., Dalian, China) according to the manufacturer’s instructions. Quantitative PCR was performed using Premix Ex Taq kit (Takara Bio Inc., Dalian, China). The average Ct was normalized to GADPH mRNA levels. 

### 2.9. Calculation of HPV DNA Copy

DNA was extracted from VIN cells with TIANamp Genomic DNA Kit (Tiangen). pUC19-HPV18 E2/E7 was used to generate standard curves. The primer set of HPV18 E7(fl) was used for quantitative PCR. HPV18 DNA copies were calculated according to the standard curve as described previously [21].

### 2.10. DNA Damage Response and Western Blotting Analysis

After treatment with 0.5 nM actinomycin D (Act D) for 24 h, cells were collected with RIPA buffer (Beyotime, Beijing, China). Protein concentration was measured with a BCA Protein Assay Kit (Beyotime, Beijing, China). Western blotting analysis was carried out with the primary antibody against P21, P53 (as listed in Table 2). 

### 2.11. Hematoxylin-Eosin (H&E) Staining

ALI 3D cultures were fixed in 4% paraformaldehyde, dehydrated, paraffin embedded, and sectioned using standard histological procedures. The sections were stained with hematoxylin and eosin (H&E) (Zhongshan Golden Bridge Company, Beijing, China) and photographed under the EVOS microscope (ThermoFisher Scientific).

### 2.12. DAB Staining

ALI or matrigel 3D cultures were fixed in 4% paraformaldehyde, embedded in paraffin, and cut into 4 μm sections. DAB staining was performed using EliVision Super DAB kit (Maixin biotech company, Fuzhou, China) as described previously [22].

### 2.13. Immunofluorescence Assay

VIN cells were cultured in 12-well plates and fixed in 4% (*w*/*v*) paraformaldehyde for 10 min and permeabilized with 0.5% Triton-X-100 for 10 min.Then the slides were probed with the primary antibodies (listed in Table 2). ALI 3D cultures were collected at indicated days and fixed in 4% paraformaldehyde, embedded, and sectioned. The slides were deparaffinized, rehydrated, and rinsed with tap water. The slides were put in 0.01 M sodium citrate (pH 6.0) at 95 °C for 15 min for antigen retrieval and then in 3% H_2_O_2_ for 10 min at room temperature. After blocking with 10% bovine serum albumin for 1 h at room temperature, the slides were probed with primary antibodies (as listed in Table 2) at 4 °C over night. DAPI (0.5 mg/mL, Wuhan Kerui Biology) was used to stain the nucleus. Then, the fluorescence was photographed under a microscope (Leica DM4000B) or laser confocal scanning microscope (Leica SP8).

### 2.14. Air-Liquid Interface (ALI) 3D Culture

ALI 3D cultures were carried as described previously [18,22]. In brief, VIN cells (2.5 × 10^5^) were suspended in 400 μL growth medium (CELLnTEC Advanced Cell Systems AG, Stauffacherstrasse, Bern, Switzerland) and seeded into the Millicell PCF inserts (12 mm size, Millipore). Cells were cultured at 37 °C and 5% CO_2_ for 72 h. The growth medium was replaced with differentiation medium (CELLnTEC Advanced Cell Systems AG) for 30 h, and then ALI 3D cultures were cultured for another 14 to 20 days and harvested at the indicated days.

### 2.15. Viral Particle Visualization by Transmission Electron Microscopy

ALI 3D cultures were collected at indicated days and fixed in transmission electron microscope (TEM) sample buffers provided by the Center for Medical Biological Structures of Wuhan University. Serial ultrathin sections of ALI 3D cultures were prepared, and images were photographed by TEM (Hitachi HT7700, Tokyo, Japan).

### 2.16. Statistical Analysis

The statistical analysis and plotting of the data were carried outwith GraphPadPrism8.0. All the experiments were performed at least three times. Data are presented as mean ± SEM, and statistical significance was considered at *p* < 0.05. (* *p* < 0.05, ** *p* < 0.01, *** *p* < 0.001).

## 3. Results

### 3.1. Establishment of Human Vulvar Intraepithelial Neoplasia (VIN) Cell Line

**Figure 1:**
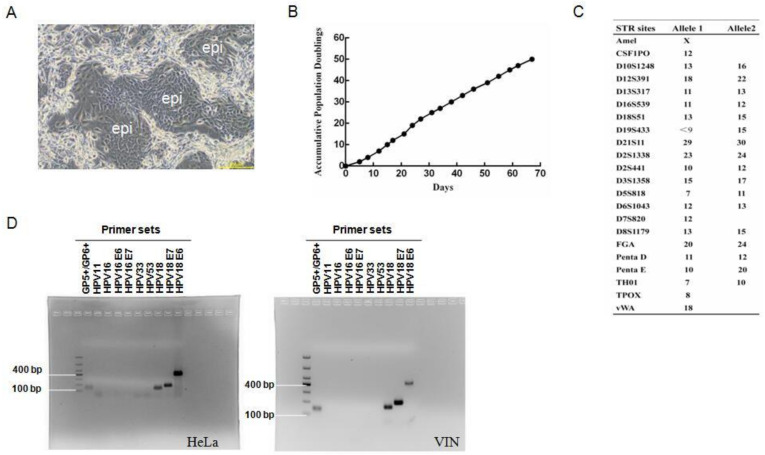
Establishment of vulvar intraepitheilial neoplasia (VIN) cells with naturally infected HPV genome. (**A**) The morphology of VIN cells (co-culture with feeder cells). The morphology image was taken with the phase contrast microscope. “epi” indicates epithelial/VIN cells. Scale bar: 200 μm. (**B**) The growth curve of VIN cells. The numbers of VIN cells were counted for each passage, and a plot of accumulated population doublings (PDs) versus growth days was constructed. (**C**) The short tandem repeat (STR) analysis of VIN cells. STR analysis of 21 loci plus the X-chromosome-specific Amelogenin locus was carried out. (**D**) HPV typing. DNA was isolated from cells, and PCR amplification was carried out using the specific primer sets listed: GP5+/GP6+ (lane 1, amplicon size 150 bp); HPV11 (lane 2, amplicon size 80 bp); HPV16 (lane 3, amplicon size 499 bp); HPV16 E6 (lane 4, amplicon size 453 bp); HPV16 E7 (lane 5, amplicon size 252 bp); HPV33 (lane 6, amplicon size 211 bp); HPV53 E6 (lane 7, amplicon size 263 bp); HPV18 (lane 8, amplicon size 172 bp); HPV18 E7 (lane 9, amplicon size 212 bp); HPV18 E6 (lane 10, amplicon size 439 bp). HeLa cells were set as a positive control (left panel). DNA Ladder used is DL 1000.

The biopsies of vulvar intraepithelial neoplasia (VIN) were collected and isolated for primary cell culture as described in Materials and Methods. Initial culture was established with irradiated mouse fibroblast 3T3 (feeder) cells in defined CR culture medium [14,15]. The morphology of VIN cells co-cultured with feeder cells was shown in Figure 1A. VIN cells were proliferated rapidly and accumulated to 50 population doublings (PDs) in 67 days (Figure 1B). Cell ID (short tandem repeats, STR) analysis was conducted to detect 22 highly polymorphic loci and verified the uniqueness of VIN cells in the database (Figure 1C). Regular PCR was performed for subtyping with general primers and HPV 11, 16, 33, 53, and 18 subtype-specific primers (as listed in Table 1). The results showed amplicons with general primers (GP5+/GP6+) and HPV18-specific primers, indicating that VIN cells carried HPV18 DNA (Figure 1D). As far as we know, this is thefirst cell line established from human vulvar intraepithelial neoplasia.

### 3.2. Different Biological Characteristics in VIN Cells Compared to HPV18 Positive Cancer Cell Line

**Figure 2:**
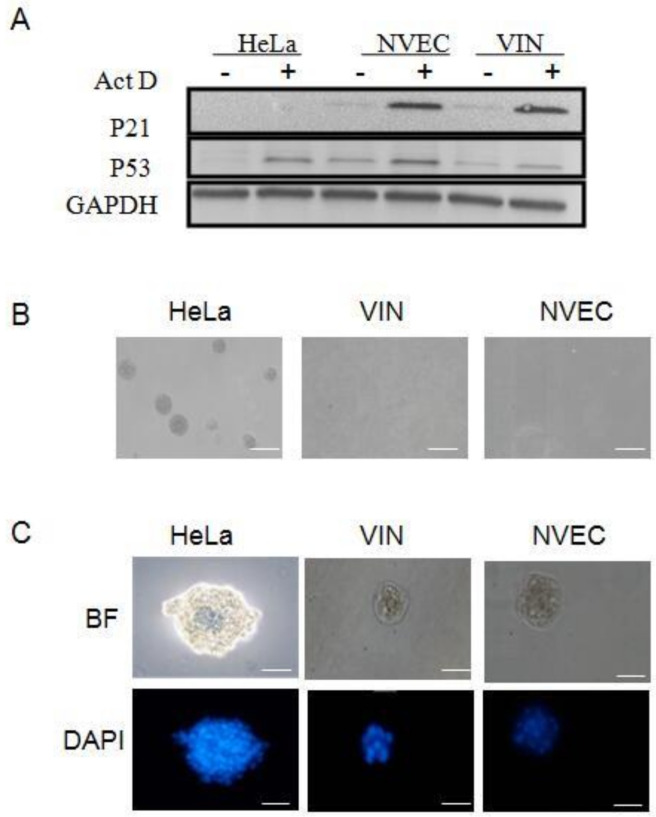
The biological differences between HPV18-positive VIN cells and HeLa cells (a cervical cancer cell line with integrated HPV18). (**A**) DNA damage response. VIN cells were treated with 0.5 nM actinomycin D (ActD) for 24 h. Cell lysate was collected. The DNA response was measured as the levels of p53 protein and its downstream target p21 with Western blotting analysis. GADPH is the loading control. HeLa cells and normal vulvar epithelial cells (NVEC) cells were used as control cells. (**B**) Anchorage-independent growth. Single-cell suspensions of cells were seeded in soft agar. HeLa cells and NVEC cells were set as positive and negative control cells, respectively. HeLa cell colonies were observed and photographed after 14 days of culture. VIN and NVEC cells were cultured and photographed after 30 days. Scale bar, 50 μm. (**C**) Cell morphology in 3D matrigel cultures. Single-cell suspension of cells was cultured in medium containing 2% matrigel for 7 days. HeLa cells and NVEC cells were positive and negative control cells, respectively. The morphology of 3D matrigel cultures was stained with 0.5 μg/mL DAPI and photographed with a fluorescence microscope. Scale bar, 50 μm.

The tumor suppressor gene p53 functions to block abnormal cells that have suffered genetic damage and prevent abnormal proliferation. In response to DNA damage, normal cells possess the ability to up-regulate the expression of p53 and in turn activate the transcription of downstream effector p21, which arrests the cell cycle in G1 phase and inhibits abnormal cellular proliferation [23,24]. To investigate the biological characteristics of VIN cells, we analyzed the DNA damage response to actinomycin D (Act D) treatment (Figure 2A). The results showed that NVEC (normal cells) increased the expression of both p53 and p21 after Act D treatment. In contrast, HeLa (tumor cells) occasionally increased the expression of p53 but not the downstream effector p21 and lost the ability to inhibit abnormal proliferation. Compared with NVEC and HeLa, VIN cells had the intact p53-mediated growth-related pathways and functions to respond to the DNA damage (Figure 2A). Next, we analyzed the transforming property of VIN cells using anchorage-independent growth assay (soft agar). As shown in Figure 2B, colonies were observed with HeLa cells after 7 days culture in soft agar. VIN cells, the same as normal NVEC, did not form colonies in soft agar even after 30 days of culture (Figure 2B). Since matrigel contains multiple growth factors, maintaining normal homeostasis and tissue morphology [19], matrigel three-dimensional (3D) culture has been widely used to evaluate differentiation potential of cells in vitro [14,16]. As shown in Figure 2C, compared with HeLa cells (irregular-sphere aggregates), VIN and NVEC cells were similar in that they formed normal differentiated, well-organized, and smooth spheres in matrigel 3D cultures. Taken together, our results demonstrated that VIN cells maintained very different biological characteristics and differentiation potential in vitro long-term cultures compared with the HPV18-positive HeLa cancer cells.

### 3.3. Squamous Epithelium and Viral Specific Markers in VIN Cells

**Figure 3:**
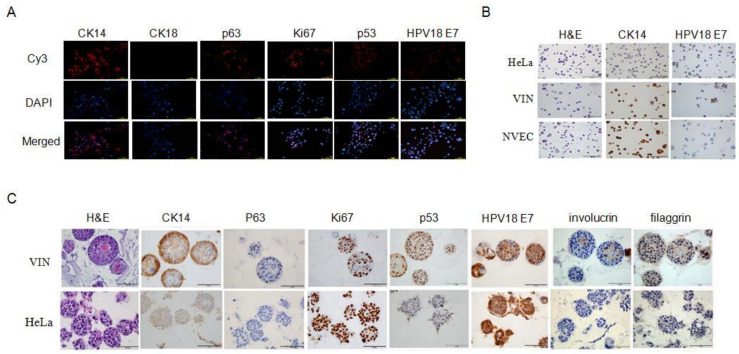
Expression of cell type-specific and viral markers in VIN cells. (**A**) Expression of cell type-specific markers in VIN cells under 2D cultures. VIN cells were seeded in 48-well plates and collected after 24 h, then fixed in 4% paraformaldehyde and detected by immunofluorescence staining with primary antibodies against CK14, CK18, p63, Ki67, p53, and HPV18 E7. Scale bar, 100 μm. (**B**) Expression of cell type-specific and viral markers in VIN, HeLa and NVEC under 2D cultures. Cells were fixed and detected by DAB staining with primary antibodies against CK14 and HPV18 E7. The results of H&E and DAB staining were photographed under a microscope. Scale bar, 100 μm. (**C**) Expression of cell type-specific and viral markers under 3D matrigel cultures. Single-cell suspensions of VIN cells and HeLa cells were cultured in medium containing 2% matrigel for 10 days. The 3D cultures were fixed with 4% (*w*/*v*) paraformaldehyde, paraffin-embedded, sectioned using standard histological procedures, and detected by DAB staining with the primary antibodies against CK14, p63, Ki67, HPV18 E7, p53, involucrin, and filaggrin. The results of H&E and DAB staining were photographed under a microscope. Scale bar, 100 μm.

To verify the origin of the VIN cells, we analyzed the expression of squamous epithelium specific markers using immunofluorescence and DAB staining. The cytokeratin (CK) expression was positive for CK14 and negative for CK18 (Figure 3A), indicating that VIN cells originated from the stratified squamous epithelium. The stemness marker p63 has been shown as a specific marker for CR cells [16]. VIN cells were established using CR method and stained positive for p63 (Figure 3A). Ki67, a marker of cell proliferation, also stained positive (Figure 3A), indicating that VIN cells possess the ability of proliferation under CR condition. The expression of HPV18 E7 and p53 was stained positive in the majority of VIN cells, while NVEC was stained negative for HPV18 E7 (Figure 3A,B). We also analyzed the expression of the above markers in matrigel 3D cultures of VIN cells. H&E staining showed the normal differentiated structure of VIN cells but not of HeLa cells in matrigel 3D cultures (Figure 3C). As an adenocarcinoma cell line, HeLa cells do not express squamous epithelium specific marker CK14 in 3D matrigel culture (Figure 3C). HeLa cells were stained negative for P63, P53, involucrin (early differentiation marker), and filaggrin (late differentiation marker) but strongly positive for Ki67 and HPV18 E7 (Figure 3C). These results demonstrated that HeLa cells lost the normal biological function and differentiation potential and gained malignant proliferation. In contrast, VIN cells were positive for CK14 and partially positive for P63, Ki67, p53, HPV18 E7, involucrin, and filaggrin in 3D matrigel culture (Figure 3C), indicating that VIN cells maintained the cell type-specific differentiation potential without malignant proliferation (Figure 2B and Figure 3C).

### 3.4. Complex Karyotypes of VIN Cells

**Figure 4:**
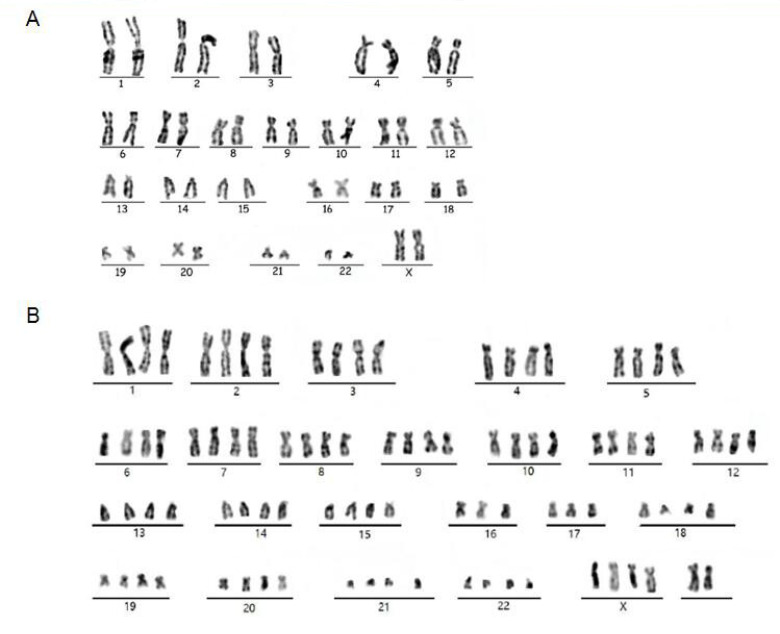
Karyotype analysis of VIN cells. The representative karyotypes of diploids (**A**) and tetraploids (**B**) of VIN cells. At least twenty metaphase spreads for VIN cells were counted and photographed under the microscope.

We have shown that VIN cells contained HPV18 DNA (Figure 1D) but did not have the ability of anchor-independent growth (Figure 2B). VIN cells maintained different biological characteristics and differentiation potential from HeLa cells (Figure 2A,C). A previous study showed that conditionally reprogrammed cells (CRCs) are karyotype stable [14]. Next, we performed the karyotype analysis of VIN cells. The results showed that both diploid (Figure 4A) and tetraploid (Figure 4B) karyotypes were observed with VIN cells. More than 70% of VIN cells were observed as diploid, which is in accordance with the biological characteristics of VIN cells. The minority of tetraploids in VIN cells may result from persistent HPV18 infection and the expression of viral oncoproteins.

### 3.5. Episomal HPV18 Genome in VIN Cells

**Figure 5:**
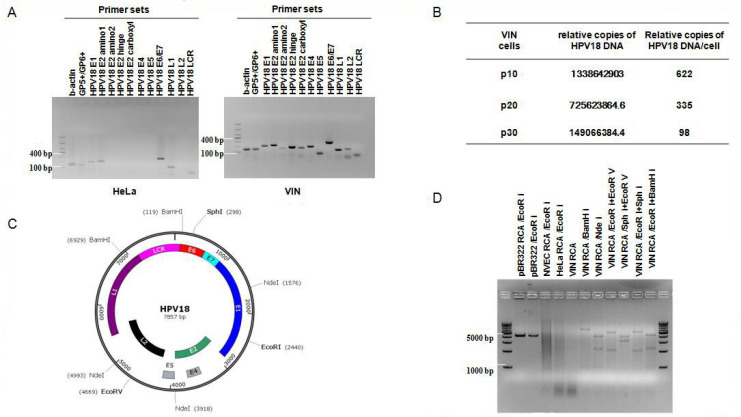
Identification of HPV18 DNA and viral genes in VIN cells. (**A**) Identification of HPV18 viral genes in VIN cells. DNA was isolated from VIN cells. HeLa were set as control cells. The following specific primer sets were used for PCR: β-actin (lane 1, amplicon size 142 bp), GP5+/GP6+ (lane 2, amplicon size 150 bp), HPV18 E1 (lane 3, amplicon size 205 bp), HPV18 E2 amino 1 (lane 4, amplicon size 223 bp), HPV18 E2 amino 2 (lane 5, amplicon size 157 bp), HPV18 E2 hinge (lane 6, amplicon size 192 bp), HPV18 E2 carboxyl (lane 7, amplicon size 177 bp), HPV18 E4 (lane 8, amplicon size 204 bp), HPV18 E5 (lane 9, amplicon size 85 bp), HPV18 E6/E7 (lane 10, amplicon size291 bp), HPV18 L1 (lane 11, amplicon size 149 bp), HPV18 L2 (lane 12, amplicon size 165 bp), and HPV18 LCR (lane 13, amplicon size 70 bp). DNA ladder used is DL 1000. (**B**) Relative copies of HPV18 DNA/cell in VIN cells in different passages. DNA was extracted from VIN cells. A recombinant plasmid pUC19-HPV18 E2/E7 was used to generate standard curve. The primer set of HPV18 E7 was used for Q-PCR. The corresponding HPV18 DNA copies and relative copies of HPV18 DNA/cell were calculated as described in Materials and Methods. (**C**) Map of prototype of HPV18 genome and cutting sites by restriction enzymes. (**D**) Digested fragments of RCA amplification products of HPV18 DNA. DNA was isolated from VIN cells. One ug of DNA stock solution was used as template for RCA amplification. And then RCA products were digested with restriction enzymes. The digested fragments of HPV18 were loaded for agarose gel electrophoresis as following: lane 1, pBR322 RCA (EcoRI); lane 2, pBR322 (EcoRI); lane 3, NVEC RCA (EcoRI); lane 4, HeLa RCA (EcoRI); lane 5, VIN RCA (no enzyme); lane 6, VIN RCA (BamHI); lane 7, VIN RCA (NdeI); lane 8, VIN RCA (EcoRI + EcoR V); lane 9, VIN RCA (SphI + EcoRⅤ); lane 10, VIN RCA (EcoRI + SphI); lane 11, VIN RCA (EcoRI + BamH I). DNA Ladder used was 1 Kb DNA Marker.

Subtyping analysis of VIN cells have shown the replicons with HPV18-specific primers same as HeLa cells (Figure 1D). As we know, HeLa is a cervical cancer cell line with integrated HPV18 DNA in the cellular genome. To investigate the status of the HPV18 genome in VIN cells, we amplified all of the HPV18 early and late genes using regular PCR (primer sets listed in Table 1). The results showed all the right sizes of amplified products from the HPV18 genes: HPV18 E1, E2 amino1, E2 amino2, E2 hinge, E2 carboxyl, E4, E5, E6/E7, L1, L2, and LCR (Figure 5A). In contrast, replicons of HPV18 E2 amino2, E2 hinge, E2 carboxyl, E4, E5, and L2 were not observed in HeLa cells (Figure 5A), indicating that the physical status of HPV18 genome in VIN cells is different from that in HeLa cells.

HPV integration to the host genome frequently occurs with E1 or E2 disruption, resulting in suppression of E2 transcription, and the integrity of E2 is taken as a marker of viral integration [25,26]. The ratio of E2_copy_/E7_copy_ usually predicts the physical status of HPV genome in samples. If the E2_copy_/E7_copy_ ratio is close to 0, the HPV genome is regarded as pure integrated form. If the E2_copy_/E7_copy_ ratio is ≥ 1 or between 0 and 1, the episomal HPV genome exists in samples [25,26]. To further define the physical status of HPV18 genome in VIN cells, we carried out quantitative PCR (qPCR) and calculated the ratio of HPV18 E2_copy_/E7_copy_. The results showed that the E2_copy_/E7_copy_ ratio is close to 0 (excluding E2 amino1) in HeLa cells, confirming the HPV18 DNA integration in HeLa cells, while the E2_copy_/E7_copy_ ratio ≥ 1 in VIN cells, indicating the episomal form of HPV18 DNA in VIN cells (Table 3). We also conducted qPCR to determine the relative HPV18 DNA copy number/cell using pUC19-HPV18 E2/E7 as a standard plasmid. The results showed that there were 622 copies of HPV18 DNA in each VIN cell at passage 10 and viral copy number decreased in VIN cells with passaging (Figure 5B). HPV18 genome was also significantly increased or amplified when VIN cells (at different passages) were under ALI 3D cultures.

Based on the existence of episomal HPV genome, we carried out rolling circle amplification (RCA) to amplify the circular HPV18 genome as described in Materials and Methods. Based on the map of the prototype of HPV18 genome (Figure 5C), we chose the restriction enzymes or combination of restriction enzymes (Table 4) to digest RCA products. The digested fragments of HPV18 were loaded for agarose gel electrophoresis (Figure 5D). Control plasmid pBR322 is circular DNA that can be amplified by RCA. Both of pBR322 and its RCA product were digested by EcoR I and observed the same size of fragments (Figure 5D). There was no digested fragment observed in HeLa cells (integrated HPV18) or NVEC cells (HPV18 negative). All digested fragments of RCA products from VIN cells, either with single enzyme cutting or double enzyme cutting, were observed with correct sizes (Figure 5D), indicating correct circular HPV18 genome in VIN cells. RCA products from VIN cells were cloned and sent to Sangon Biotech (Shanghai, China) for sequencing. The alignment results confirmed the RCA product as the HPV18 genome sequence. Our results demonstrated that there exists episomal HPV18 DNA in VIN cells, while the integrated form of HPV18 DNA could not be excluded. 

### 3.6. Viral Life Cycle of Naturally Infected HPV18 in VIN Cells under Air-Liquid (ALI) 3D Cultures

**Figure 6:**
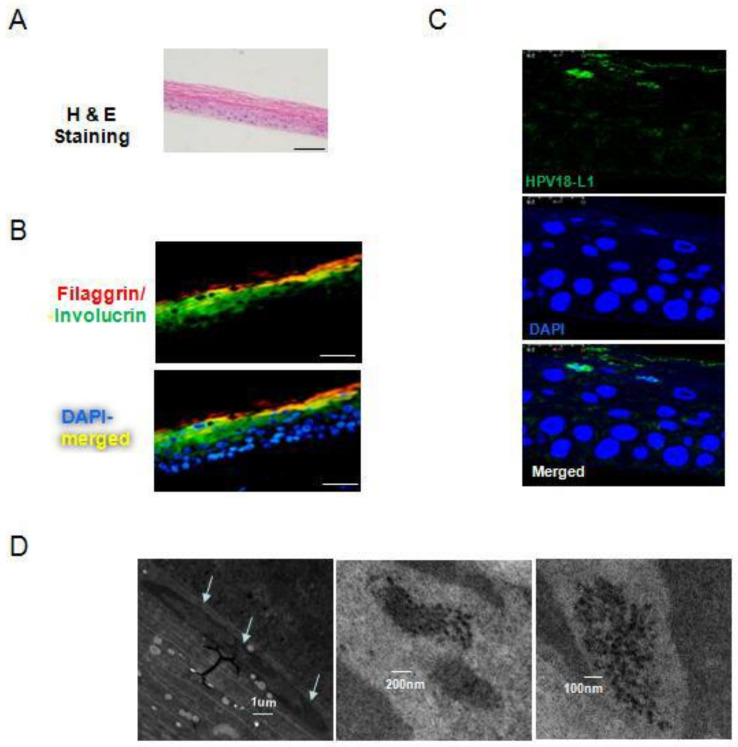
Differentiation-dependent expression of HPV18 late protein and viral particle assembly under ALI 3D cultures. (**A**) H&E-stained section of VIN cell-derived ALI 3D culture. ALI 3D culture of VIN cells was harvested on day 18 and fixed with 4% (*w*/*v*) paraformaldehyde, paraffin embedded, and sectioned. The images of H&E staining were photographed. Scale bar, 100 μm. (**B**) Co-localization of filaggrin and involucrin in VIN cell-derived ALI 3D cultures. Immunofluorescence analysis was carried out using specific primary antibodies against filaggrin and involucrin. Nuclei were stained with 0.5 μg/mL DAPI. Scale bar, 50 μm. (**C**) Immunofluoresence detection of the major capsid protein L1 in VIN cell-derived ALI 3D cultures by confocal microscope. VIN cells were seeded in the inserts for ALI 3D cultures and harvested at day 18. Then ALI cultures were fixed, paraffin embedded, sectioned, and stained with primary antibody against HPV18 L1 with immunofluoresence analysis. The nuclei were stained with 0.5 μg/mL DAPI. (**D**) Detection of HPV18 virions in VIN cell-derived ALI 3D cultures under transmission electron microscope (TEM). VIN cells were cultured in ALI and harvested at day 18. Then ALI cultures were fixed, and ultrathin sections were made after staining and observed under TEM. Scale bar, 1 μm, 100 nm, 200 nm.

HPVs infect the basal layer of epithelium but replicate and complete the viral life cycle in the upper differentiated layers of epithelium [4,21,27]. Previously, we were able to reconstruct stratified vaginal epithelium with normal vaginal epithelial cells (NVEC) using air–liquid interface (ALI) 3D cultures [18]. NVEC-derived ALI 3D culture had a similar histological structure to that of vaginal tissue. To investigate the life cycle of naturally infected HPV18 in VIN cells, we carried out ALI 3D cultures with VIN cells. The histology sections of ALI 3D culture were stained with H&E and showed the stratified layers of epithelium (Figure 6A). We also determined the expression and localization of involucrin (early differentiation marker) and filaggrin (late differentiation marker) in ALI 3D cultures. The immunofluoresence results validated the well-differentiated epithelium structure of VIN cells-derived ALI 3D culture, showing involucrin expressed in the spinous and granular layers and filaggrin expressed mainly in the cornified layer of 3D culture (Figure 6B) [16,23]. We expected that these ALI 3D cultures would mimic a differentiated epithelial environment for HPV18 to initiate viral genome replication and the expression of early and late viral proteins. L1 is the major capsid protein of HPVs and also a marker of viral productive amplification, and these are essential for virion formation and transmission [2,28]. The vegetative amplification of the HPV life cycle requires stringent differentiated epithelium; however, the expression of L1 and progeny viruses was not observed in raft cultures of two previously established cell lines from cervical intraepithelial neoplasia (CIN), W12 and CIN612 [11,29,30]. Interestingly, immunofluorescence staining showed the expression of L1 in the cornified layer of ALI 3D cultures of these new VIN cells (Figure 6C). More importantly, we observed progeny viral particles in the ALI 3D cultures using transmission electron microscope (TEM). Similar to the location of L1 protein, these viral particles were only observed in the cornified layer with electron density and formed paracrystalline arrays (Figure 6D). Our results demonstrated that HPV18 initiated viral genome amplification and fulfilled viral life cycle under ALI 3D cultures, providing a novel physiological/pathological model for studies of naturally infected HPV life cycle, virus–host interaction, and cancer initiation.

## 4. Discussion

HPVs infect human mucosal or cutaneous epithelium [28,31]. HPV types are further classified into low-risk (LR) HPVs and HR-HPVs based on their associations with benign and malignant diseases [31]. In addition to tissue tropism, HPVs also differ in their clinical manifestations [2]. HPV infection initially occurs to the self-renewing basal cells of a stratified epithelium through micro-abrasion or wound. Then, HPVs maintain very low copies of viral genome and express essential early viral proteins in basal layer cells [28]. Upon the host cell differentiation of the stratified epithelia, HPVs start viral genome amplification, express late genes, and produce progeny viruses [2,32]. At each step, viruses interact with host cells and hijack cellular signaling pathways and machinery to facilitate the HPV life cycle and evade the host immune system as well. The life cycles of different HPV types may be very different due to anatomical variations in infected epithelia and microenvironments [2,28]. Therefore, it is of great importance to establish cell lines from anatomically distinct lesions and cancers to investigate the molecular mechanisms by which different types of HPVs interact with host cells for persistent infection, transformation of host cells, and cancer initiation and progression. However, primary cell culture from patient-derived lesions or cancers has been extremely challenging for decades. So far, there are only two naturally HPV-infected cell lines (W12 and CIN612) from CIN lesions [11,12] and few HPV16 and HPV18-positive cervical cancer cell lines (HeLa, SiHa, Caski, etc.). In the current study, we were able to establish the first human cell line from naturally HPV18-infected vulvar lesions.

Previous studies reported HPV amplification as episomal DNA in low-grade lesions of patients, while HPV was often found integrated into the host genomes in high-grade lesions or cancers [3]. Both W12 and CIN612 were established from CIN I lesions and contained episomal HPV16 DNA and HPV31b, respectively [11,12]. These new VIN cells were isolated from high-grade vulvar lesion and stably carried episomal HPV18 genome. Karyotype analysis showed complex karyotypes with majority diploids in the VIN cells; 30% of VIN cells with tetraploid karyotypes may be due to HPV replication and/or expression of viral oncoproteins. However, compared with the HPV18-positive cancer cell line HeLa, these new VIN cells maintained their ability to respond to DNA damage signaling and their differentiation potential. These VIN cells also failed to form colonies in soft agar assay. Thus, these cells would be valuable for virus life cycle and persistence, virus–host interactions, especially innate immune responses, and cancer initiation as well. It has been shown that CR cells maintain the same biological characteristics as their originating tissue [14,16,22]. Our results suggested that even high-grade intraepithelial neoplasia in vivo may not have tumorigenicity and may regress even with the natural course of HPV infection. We expect that cervical conization surgery can be completely or partially avoided if effective anti-HPV drugs were developed for HPV-associated lesions. In terms of VIN cells with naturally infected HPV18, it would be extremely important to evaluate whether these cells can serve as a novel platform for anti-HPV drug discovery.

As we discussed above, primary HFKs have been used instead of female genital tract samples due to the availability of tissue specimens. HPV DNAs were frequently transduced into HFKs to study HPV biology and oncogenesis [33,34]. Since the HPV life cycle strictly depends on epithelial differentiation, HPV cannot be propagated in two-dimensional (2D) cultures. Raft culture or ALI 3D culture were developed to model a differentiation microenvironment for amplification of HPV genome [30,35]. However, the recapitulation of productive life cycle in vitro has proven very difficult. Long-term virus–host interactions, especially the cell proliferation/differentiation manipulated by the interactions, become at high risk for the transformation of the host cells, which is not a primary intent of HPV infection [2,28,31]. E5, E6, and E7 from HR-HPVs play critical roles in the transformation of host cells [36,37,38,39,40]. There are several cancer cell lines with HPV16 or HPV18; keratinocyte system (immortalized HaCat cells or primary keratinocytes) with the transfection of HPV genomes [41,42]; keratinocytes (immortalized HaCat cells or primary keratinocytes) infected with quasivirus; psendovirus; “native” HPV particles [43]; and immortalized cell lines with E6/E7, SV40, and hTERT. There are also two or three patient-derived cells, for example, W12 (HPV16) and 9E (HPV31) [44]. To date, many precise processes of HPV life cycle and oncogenesis remain largely unknown due to lacks of natural systems for productive and abortive HPV life cycles [36,37,38,39,40,44]. The studies on HPV biology, cancer initiation, and the development of HPV- or cancer-targeting therapies have been significantly restrained by the lack of in vitro and in vivo biological models.

HPV DNA-transduced HFKs could hardly initiate de novo productive infections in raft cultures [3,45]. Later, a Cre-loxP-mediated recombination system was created to produce autonomous HPV18 genomes in HFKs by raft culture [45]. Most of the above systems are questioned on relation with in vivo lesion since they were generated bytransduction with exogenous HPV18 genomes and HPV life cycle could only be observed with high-multiplicity infection of HPVs [3]. Thus, naturally HPV-infected cell lines from anatomically distinct lesions and cancers are still urgently needed. 

CR can establish long-term cultures from a variety of human epithelial specimens, including normal tissue, precancerous lesions, and cancer [15]. CR reprograms all primary cell populations to an adult stem cell-like status instead of clonal selection [16]. Therefore, we could not rule out the possibility that the cell line contained some normal cells if there were normal cells in the vulvar biopsy. Moreover, CR cells maintain tissue-specific differentiation potential and can differentiate when the CR condition is removed [14,16,17,46]. This feature of CR cells is critical for establishing a pathophysiological model with natural HPV-associated lesions. Our results showed the VIN cells formed a well-differentiated structure of a stratified epithelium with in situ expression of involucrin and filaggrin under ALI 3D culture. This 3D mimic-differentiated epithelium is essential for a productive HPV life cycle since viral copy number was not increased in calcium-induced differentiated monolayer VIN cells. Our data cannot rule out the possibility that there are mixed episomal and integrated HPV genomes in VIN cells; however, only episomal HPV18 in VIN cells can initiate productive amplification, assemble mature virions, and complete the viral life cycle in the VIN-derived ALI 3D cultures. This first cell line from human vulvar lesions with episomal HPV DNA provides a valuable model for studying HPV biology and HPV-associated cancer.

## 5. Conclusions

In conclusion, we established the first human cell line from vulvar lesions using the CR approach. These new VIN cells maintained different biological characteristics from and did not have transforming properties in vitro compared with the HPV18-positive cervical cancer cell line HeLa. VIN cells contained naturally infected episomal HPV18 genome. The viral late gene L1 was expressed and progeny viral particles were assembled when VIN cells were cultured under ALI 3D. VIN cell-derived ALI 3D culture provides a valuable model for HPV biology studies, HPV-associated cancer initiation and progression, and drug-screening platforms.

## Figures and Tables

**Table 1:** List of PCR primers used in this study.

Primer Names	Sequences (5’-to-3’)	Amplicon Size (bp)
GP5+/GP6+-F	TTTGTTACTGTGGTAGATACTAC	150
GP5+/GP6+-R	GAAAAATAAACTGTAAATCATATTC	
HPV18-F	CCGAGCACGACAGGAACGACT	172
HPV18-R	TCGTTTTCTTCCTCTGAGTCGCTT	
HPV18 E1-F	ATGGCTGATCCAGAAG	205
HPV18 E1-R	ATGCCTGTGCTGTCTCTAGC	
HPV18 E2 (amino 1)-F	AGACACCGAAGGAAACCCTTT	223
HPV18 E2 (amino 1)-R	GCTTTATGTGCTTTACTTTTTGA	
HPV18 E2 (amino 2)-F	TGCAAGACACATGCGAGGAA	157
HPV18 E2 (amino 2)-R	CATGTTCCTGCATCAGTCATAT	
HPV18 E2 (hinge)-F	AAAATATGGGAACACAGGTACG	192
HPV18 E2 (hinge)-R	GCCGACGTCTGGCCGTAGGTCT	
HPV18 E2 (carboxyl)-F	TACAGGCAACAACAAAAGACG	177
HPV18 E2 (carboxyl)-R	CCTGTTTTTTCATTGCCTGC	
HPV18 E4-F	TGTGCAGTACCAGTGACGAC	204
HPV18 E4-R	GTGTAGCTGCACCGAGAAGT	
HPV18 E5-F	CCGCTTTTGCCATCTGTCTG	85
HPV18 E5-R	CTGTGGCAGGGGACGTTATT	
HPV18 L1-F	GCCGCCACGTCTAATGTTTC	149
HPV18 L1-R	CCCTGTGATAAAGGACGCGA	
HPV18 L2-F	ATGACAACCCGGCCTTTGAG	165
HPV18 L2-R	ACATAGTTGCCCGTTGACCT	
HPV18 LCR-F	AGGGAGTAACCGAAAACGGTC	70
HPV18 LCR-R	GTATTGTGGTGTGTTTCTCAC	
HPV18 E6/E7-F	ACGACAGGAACGACTCCAAC	291
HPV18 E6/E7-R	AGGTCGTCTGCTGAGCTTTC	
β-actin-F	GCACGGCATCGTCACCAACT	142
β-actin-R	CATCTTCTCGCGGTTGGCCT	
HPV11-F	CGCAGAGATATATGCATATGC	80
HPV11-R	AGTTCTAAGCAACAGGCACAC	
HPV16-F	GTCAAAAGCCACTGTGTCCT	499
HPV16-R	CCATCCATTACATCCCGTAC	
HPV16 E6-F	ATGTTTCAGGACCCACAGGA	453
HPV16 E6-R	CAGCTGGGTTTCTCTACGTGTT	
HPV16 E7-F	ATGCATGGAGATACACCTAC	252
HPV16 E7-R	CATTAACAGGTCTTCCAAAG	
HPV33-F	AACGCCATGAGAGGACACAAG	211
HPV33-R	ACACATAAACGAACTGTGGTG	
HPV53 E6-F	GGGTATCCGTATGGAGTGTGC	263
HPV53 E6-R	GTTGTGTGTCTCCAGCATGTC	
HPV18 E6-F	CGCTTTGAGGATCCAACACG	439
HPV18 E6-R	GTTCCTGTCGTGCTCGGTTG	
HPV18 E7-F	GTCACGAGCAATTAAGCGAC	212
HPV18 E7-R	CACAAAGGACAGGGTGTTCA	
GAPDH-F	CTGGGCTACACTGAGCACC	101
GAPDH-R	AAGTGGTCGTTGAGGGCAATG	
HPV18 E2 (fl)-F	GTTGTAAAACGACGGCCAGTATGCAGACACCGAAGGAA	1098
HPV18 E2 (fl)-R	TTACATTGTCATGTATCCCACC	
HPV18 E7 (fl)-F	TGGGATACATGACAATGTAAATGCATGGACCTAAGGCA	318
HPV18 E7 (fl)-R	GATCCCCGGGTACCGAGCTCTTACTGCTGGGATGCACA	

**Table 2:** List of primary antibodies used in this study.

Antibody	Species	Vendor/Source	Catalogue #
CK14	mouse	Santa Cruz Biotech	sc-23878
CK18 P21 P53 P63 Ki67 Filaggrin Involucrin Involucrin HPV18 E7 HPV18 L1	mouse Rabbit mouse Rabbit mouse mouse Rabbit mouse mouse Rabbit	Santa Cruz Biotech Abcam Santa Cruz Biotech Abcam Cell Signaling Santa Cruz Biotech Abcam Santa Cruz Biotech Santa Cruz Biotech biorbyt	sc-32329 ab-109199 sc-126 ab-124762 9449S sc-66192 ab-227530 sc-21748 sc-365035 p06794
GAPDH	mouse	Santa Cruz Biotech	sc-365062

**Table 3:** Ratio of HPV18 E2_copy_/E7_copy_ in VIN cells.

	HeLa				VIN			
Gene	Ct	Log Copies	Copies	E2/E7	Ct	Log Copies	Copies	E2/E7
E2 amino1	22.48	7.6	39,959,130.32	0.62	18.56	9.01	1,014,918,805	1.94
E2 amino2	28.75	5.23	169,033.01	0.00	18.90	8.74	555,874,925.75	1.06
E2 hinge	27.80	5.57	371,777.79	0.01	18.64	8.84	691,655,509.02	1.32
E2 carboxy1	26.95	5.90	795,092.58	0.01	18.73	8.84	693,065,846.55	1.32
E7	20.64	7.82	65,874,356.7	-	18.13	8.72	524,162,224	-

**Table 4:** Digested HPV18 DNA fragments by restriction enzymes.

Enzymes	Number of Cutting Fragments	Size of Cutting Fragment (bp)
EcoRⅠ	1	7857
BamHⅠ	2	1047, 6810
NdeⅠ	3	1075, 2342, 4440
EcoRⅠ+ EcoRⅤ	2	2229, 5628
EcoR Ⅴ+ SphⅠ	2	3486, 4371
EcoRⅠ+ SphⅠ	2	2142, 5715
EcoRⅠ+ BamHⅠ	3	1047, 2321, 4489

## Data Availability

The data of this study are available on reasonable request from thecorresponding authors.
